# Bonding formation and gas absorption using Au/Pt/Ti layers for vacuum packaging

**DOI:** 10.1038/s41378-021-00339-x

**Published:** 2022-01-17

**Authors:** Shingo Kariya, Takashi Matsumae, Yuichi Kurashima, Hideki Takagi, Masanori Hayase, Eiji Higurashi

**Affiliations:** 1 Graduate School of Science and Engineering, Tokyo University of Science, Chiba, 278-8510 Japan; 2 Device Technology Research Institute, National Institute of Advanced Industrial Science and Technology, Ibaraki, 305-8564 Japan

**Keywords:** Electrical and electronic engineering, Structural properties

## Abstract

In this study, we developed a metal multilayer that can provide hermetic sealing after degassing the assemblies and absorbing the residual gases in the package. A package without a leak path was obtained by the direct bonding of the Au/Pt/Ti layers. After packaging, annealing at 450 °C caused thermal diffusion of the Ti underlayer atoms to the inner surface, which led to absorption of the residual gas molecules. These results indicated that a wafer coated with a Au/Pt/Ti layer can provide hermetic sealing and absorb residual gases, which can simplify vacuum packaging processes in the electronics industry.

## Introduction

Vacuum packaging plays a critical role in the electronics industry, as it is necessary for maximizing performance and extending product life^1–3^. The following three processes are important for achieving and maintaining vacuum packaging:

- Degassing assemblies before packaging.
- Sealing the device substrate without leakage.
- Removing residual gases after packaging.

Generally, getter materials that can absorb reactive gases are used to eliminate residual gas molecules in a package^4–6^. In particular, thin layers that consist of reactive metals, such as Ti, V, and Zr, coated with an inactive layer, such as oxides and Au^7^, are common. They are called non-evaporative getter (NEG) films. These NEG films are deposited and patterned on the assemblies for vacuum packaging. The underlayer metal atoms diffuse into the inner surface of the package and absorb the residual gases by activating the NEG film at a high temperature after packaging^7–10^.

In addition to the NEG film, bonding layers are fabricated on cap wafers for sealing. To simplify the fabrication of the cap wafer, our research group developed a packaging process using a Au/Ti (from top to bottom) layer^11^. The Au surfaces can directly bond even at room temperature by atomic diffusion and grain growth across the bonding interface^12–15^. Thus, the Au/Ti layer can act as an NEG layer as well as a bonding layer for hermetic packaging. In this study, a cap substrate with cavities metalized with a Au/Ti film was bonded to a device substrate with a Au sealing layer in a vacuum. Subsequently, the packaged structure was annealed at 150–400 °C to cause the Ti underlayer atoms to diffuse to the surface, which caused reactions with the residual gases. As the NEG and bonding layers are simultaneously deposited and an additional patterning step is not necessary, the proposed technique simplifies the packaging process. This simple process is helpful in the microelectromechanical systems (MEMS) industry because a large part of the manufacturing costs is associated with complex packaging processes^2^.

**Fig. 1:**
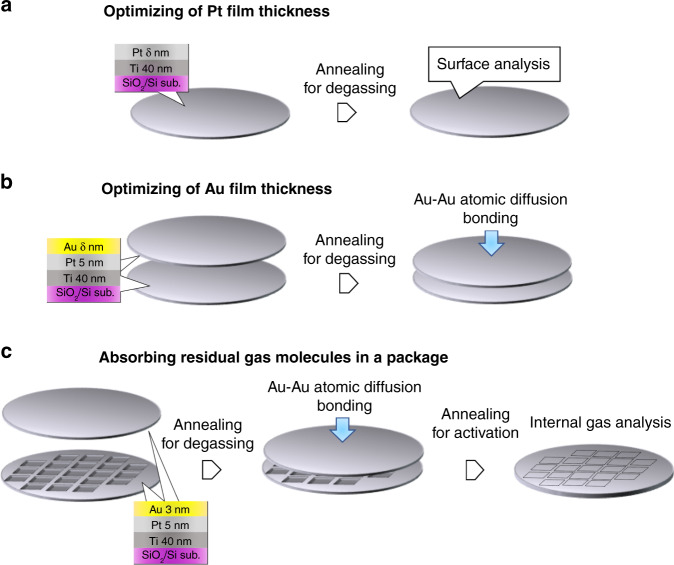
Experimental procedures. We optimized (**a**) Pt and **b** Au film thickness and **c** packaged cavities. In the bonding experiments, both substrate surfaces were metallized by Au/Pt/Ti layers

However, we found that the Au/Ti films could not form bonds after the degas annealing steps^16,17^ because Ti oxides, which struggle to form bonds, developed on the film surface even after annealing in a vacuum. This is undesirable for vacuum packaging because the molecules on the surface (i.e., H_2_O, N_2_, and hydrocarbons) could not be removed before packaging^18^. This study aims to develop a metal multilayer film that enables bond formation after the assemblies are degassed and residual gas is absorbed after packaging. We have studied the ability of the Au/Pt/Ti film to form bonding and gettering films^19,20^. The diffusion of Ti atoms can be controlled by inserting a Pt barrier layer between the Au and Ti layers^16,17^. A thick Pt layer enables bonding after degassing but possibly prevents gas absorption due to the diffusion of Ti atoms^21–23^. In the present study, the thickness of the Au/Pt/Ti multilayer was optimized, as illustrated in Fig. 1a, b. In addition, vacuum cavities were fabricated by the bonding of Si substrates coated with the optimized Au/Pt/Ti layers, as shown in Fig. 1c.

## Results

### Optimizing the Pt layer thickness

**Fig. 2:**
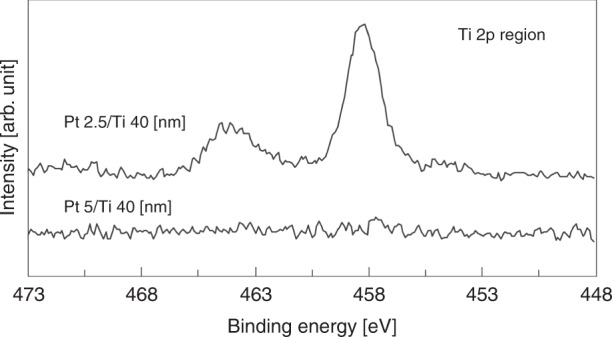
XPS spectra of the Ti 2p region for annealed Pt/Ti layers. Pt (2.5 nm)/Ti (40 nm) and Pt (5 nm)/Ti (40 nm) layer surfaces were annealed at 200 °C for 10 min

As Ti atoms rapidly diffuse into Au and prevent bonding, the Ti underlayer atoms should be blocked by the Pt layer for hermetic sealing after degassing. Figure 2 shows the XPS spectra of the Ti 2p region for the Pt (2.5 nm)/Ti (40 nm) and Pt (5 nm)/Ti (40 nm) layer surfaces after annealing at 200 °C for 10 min. The peak at 458.2 eV indicates that the Ti atoms diffused through the 2.5-nm-thick Pt layer and formed TiO_2_ on the surface. This result suggests that bonding cannot be achieved after degassing when there is a Pt barrier layer with a thickness of 2.5 nm. The TiO_2_ layer was not generated on the surface when a Pt barrier layer of 5 nm thickness was deposited. It is believed that the 5-nm-thick Pt layer on the Ti layer was a continuous film, but the 2.5-nm-thick layer was a discontinuous island structure. Thus, the thickness of the Pt layer is preferably 5 nm or more to ensure bond formation after degas annealing at 200 °C for 10 min. As a thin Pt barrier layer is required for the absorption of residual gases by the diffusion of Ti atoms, it is assumed that a 5-nm-thick Pt layer is optimal for packaging.

### Optimizing the Au layer thickness

**Fig. 3:**
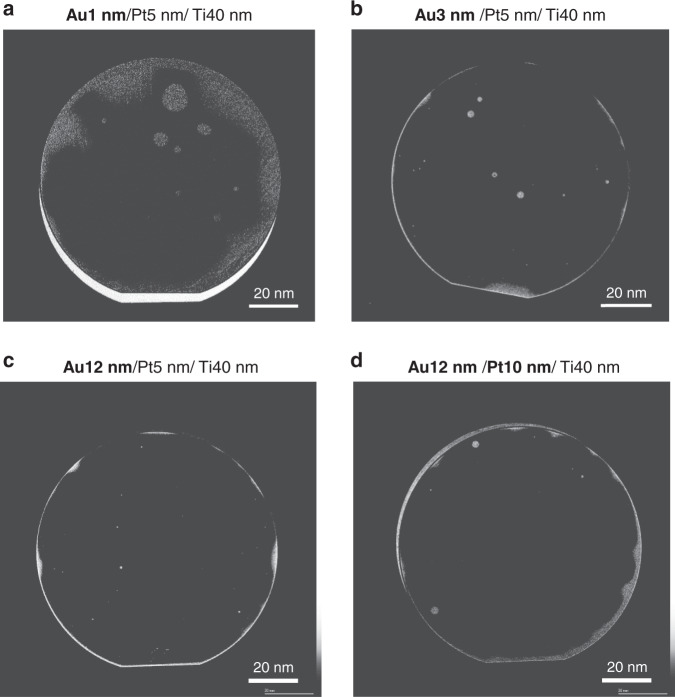
SAM images of the bonded Si substrates. They were bonded using (**a**) Au (1 nm)/Pt (5 nm)/Ti (40 nm), **b** Au (3 nm)/Pt (5 nm)/Ti (40 nm), **c** Au (12 nm)/Pt (5 nm)/Ti (40 nm), and **d** Au (12 nm)/Pt (10 nm)/Ti (40 nm) layers

**Fig. 4:**
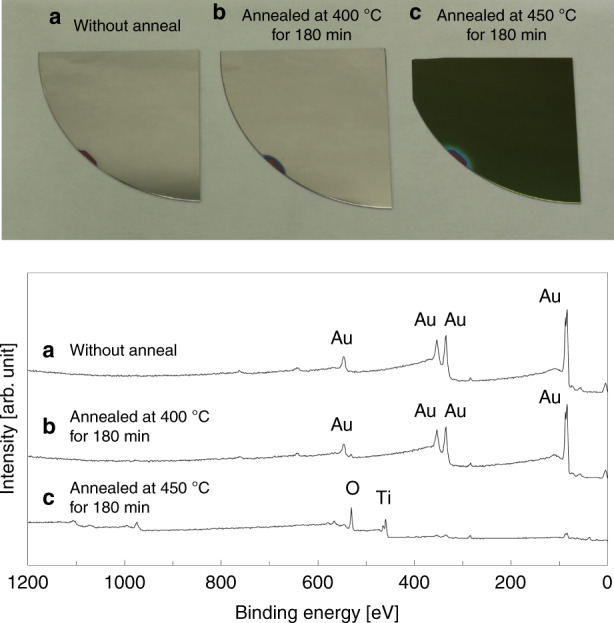
Au/Pt/Ti films before and after annealing. Specimens and XPS surface spectra of the Au (3 nm)/Pt (5 nm)/Ti (40 nm) layers (**a**) before annealing, **b** after annealing at 400 °C for 180 min, and **c** after annealing at 450 °C for 180 min

Figure 3 shows the mapping of voids between the bonded Au/Pt/Ti films observed using a scanning acoustic microscope (SAM); ultrasonic signals are reflected in the bright regions when unbonded areas are present. The Au (3 nm)/Pt (5 nm)/Ti (40 nm) and Au (12 nm)/Pt (5 nm)/Ti (40 nm) layers were successfully bonded, except for the areas where particles were present. Moreover, there were large unbonded areas near the wafer edge and in the center according to the SAM image of the bonded Au (1 nm)/Pt (5 nm)/Ti (40 nm) layers. It is believed that Pt atoms were present on the surface, as the Au layer was atomically thin in the unbonded areas. While Au surfaces can form bonds at low temperatures, Pt surfaces cannot form bonds because of the low self-diffusion coefficient^13^. In addition, another bonding experiment using a thicker film was demonstrated. As shown in Fig. 4d, the Au (12 nm)/Pt (10 nm)/Ti (40 nm) films were successfully bonded. Consequently, we determined that the Au layer was required to be 3 nm thick or greater for bonding after the degassing annealing step. Furthermore, the bonding strength of these bonded specimens was high, as all specimens had tensile strengths over 10 MPa.

### Absorbing gas molecules in air

Figure 4 shows the specimens and XPS surface spectra of Si substrates coated with Au (3 nm)/Pt (5 nm)/Ti (40 nm) layers. These samples were annealed at different temperatures. The appearance of the wafer annealed at 400 °C for 180 min is similar to that before annealing, whereas that of the wafer annealed at 450 °C for 180 min clearly changes. This result seems to be because of the interference color developed due to the formation of a Ti oxide layer on the surface. These results are consistent with the XPS surface analysis, which suggests that the surface of the wafer annealed at 400 °C for 180 min was the same as that before annealing; however, a Ti oxide layer was formed on the surface after annealing at 450 °C for 180 min.

**Fig. 5:**
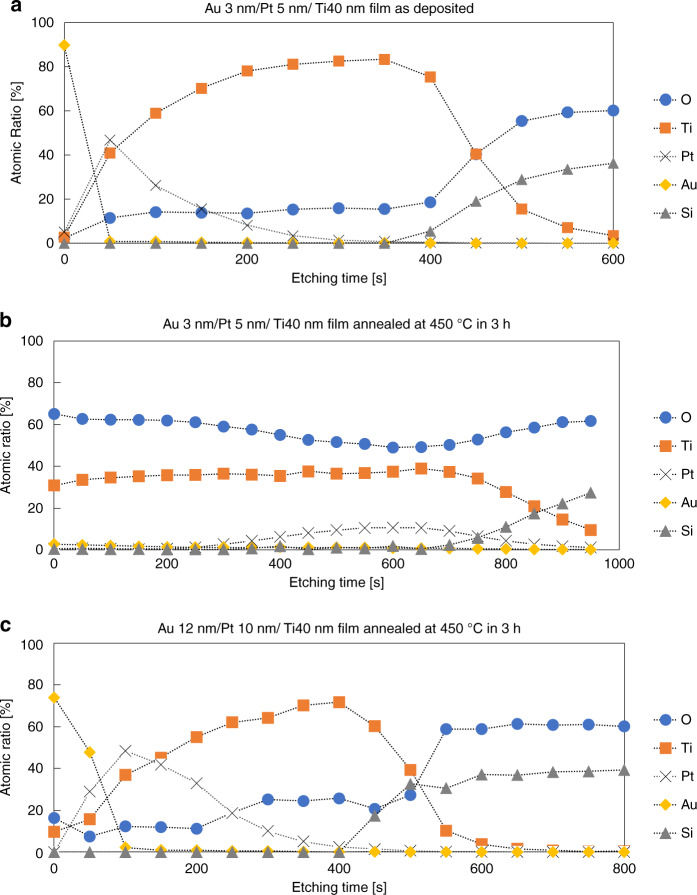
Composition depth profiles of the Au/Pt/Ti films. Au (3 nm)/Pt (5 nm)/Ti (40 nm) layer (**a**) before and **b** after activation annealing at 450 °C for 180 min in atmospheric air. **c** Au (12 nm)/Pt (10 nm)/Ti (40 nm) layer annealed at 450 °C for 180 min

The depth profiles of the Au/Pt/Ti films before and after the gas absorption process were investigated using XPS, as shown in Fig. 5a–c. The ion gun used for depth analysis has an etching rate of ~1 nm/10 s for SiO_2_. Notably, the presence of oxygen atoms in the deposited film is assumed to be because of absorption by Ti atoms in the XPS apparatus. Before annealing, the surface consisted mainly of Au; however, the annealed multilayer consisted mainly of O and Ti. The atomic ratio of Ti:O in the Au (3 nm)/Pt (5 nm)/Ti (40 nm) layer was approximately 1:2 at the surface; however, the number of O atoms decreased in the deeper region. Thus, it is assumed that the oxygen atoms were absorbed by the Ti atoms on the surface and diffused into the multilayer. Au atoms were present in the near-surface region; however, the Pt atoms moved to the bottom of the layers. In the Au (12 nm)/Pt (10 nm)/Ti (40 nm) layer, however, the number of Ti and O atoms at the surface was small, as shown in Fig. 5c. This indicates that thin Au and Pt layers are desirable for efficient gas absorption.

### Absorbing residual gas molecules in package

**Fig. 6:**
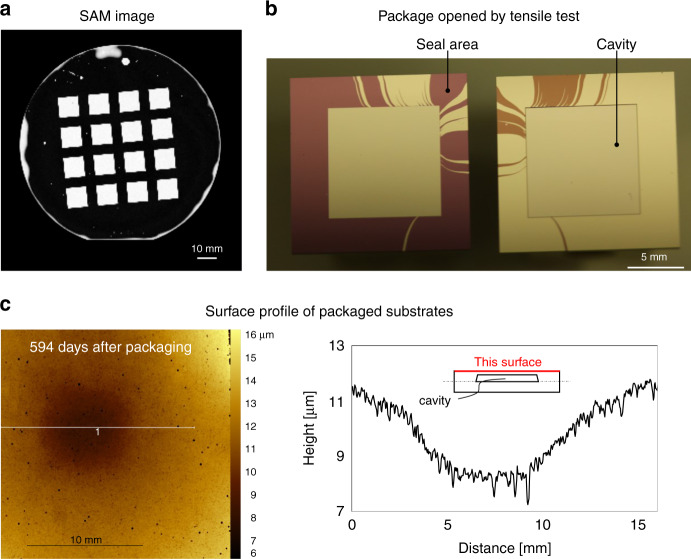
Investigation of packaged cavities. **a** SAM image of the packaged structures fabricated using the Au (3 nm)/Pt (5 nm)/Ti (40 nm) bonding layers. **b** Package opened by a tensile tester. **c** Optical surface profile of packaged substrates

The substrate with cavities and the flat substrates coated with Au (3 nm)/Pt (5 nm)/Ti (40 nm) layers were bonded in a vacuum after degassing. Figure 6a shows the SAM image of the bonded specimen, wherein bright 10-mm-square regions formed because of the packaged structures. In addition to these structures, some tiny unbonded areas were observed around the edges. The edges were assumed to be contaminated when the substrates were held by the metal deposition machine and tweezers. Otherwise, other areas were mostly bonded; thus, the cavities were tightly sealed without leak paths.

After activation annealing at 450 °C for 180 min, one of the packages was subdivided using a dicing machine and fractured by a tensile tester. The specimen fractured when a tensile strength of 7.31 MPa was applied. As shown in Fig. 6b, in the fractured specimen, the interface between the Ti and SiO_2_ surfaces was broken instead of the Au/Au bonding interface.

Figure 6c shows the surface profile of the package. Note that the observation was performed 594 days after vacuum packaging. The 10 mm square concave shape revealed that the internal vacuum was maintained. In addition, a fine leak test was performed using He gas for the bonded substrates with and without the cavity (the package structure is shown in the supplemental material). There was no significant difference in the amounts of detected He gas, as listed in Table 1. These results indicate that the gas leakage through the bonded area was limited).

**Fig. 7:**
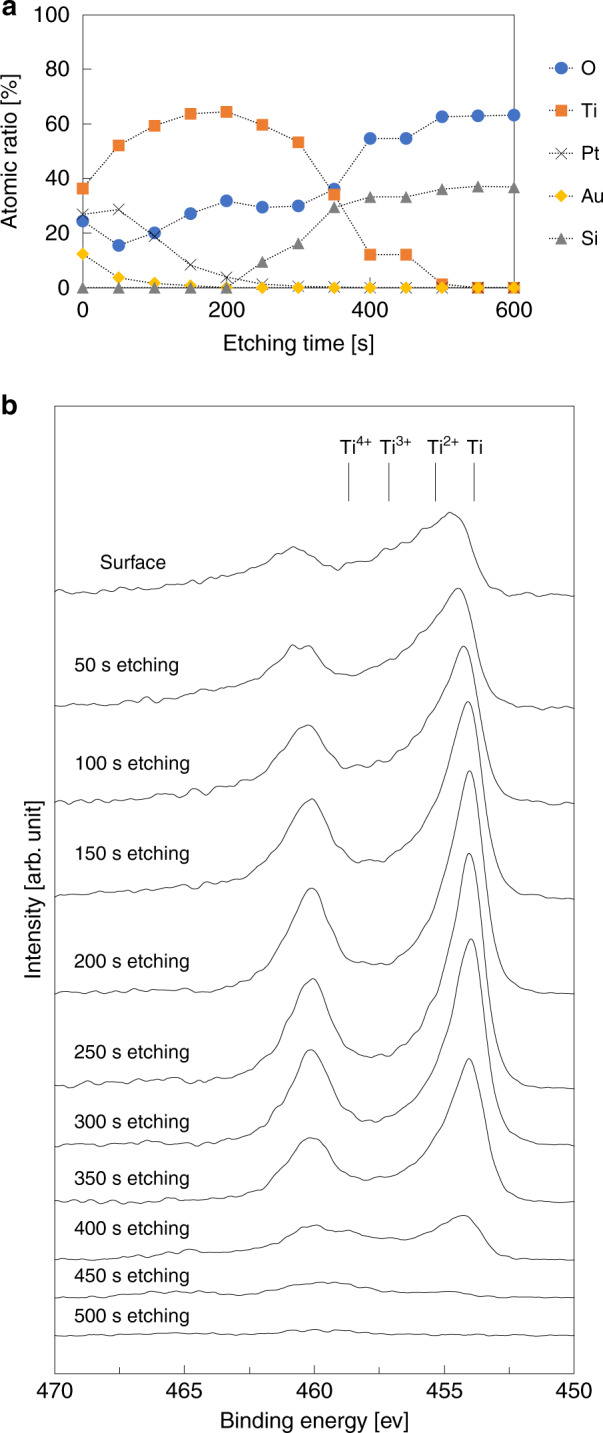
Chemical composition of Au/Pt/Ti film annealed in package. **a** Composition depth profiles of the Au (3 nm)/Pt (5 nm)/Ti (40 nm) layer after activation annealing at 450 °C for 180 min in the packaged area between substrates. **b** XPS spectra of the Ti 2p region for the Au (3 nm)/Pt (5 nm)/Ti (40 nm) layer annealed at 450 °C for 180 min in the packaged environment

The surface chemical composition of the inner package was investigated using XPS, as shown in Fig. 7a. The Ti atoms reached the surface by high-temperature annealing. The ratio of O compared with Ti was lower than that of the specimen annealed under atmospheric conditions (Fig. 5) because the number of gas molecules in the package was limited. Figure 7b shows the XPS spectra of the Ti 2p region at each etching step. The depth profile and spectra revealed that the Ti atoms in the near-surface region reacted with the residual gases; however, the inside region of the multilayer remained unreacted. Thus, it is highly expected that the metal multilayer can absorb more residual gas by additional annealing.

**Fig. 8:**
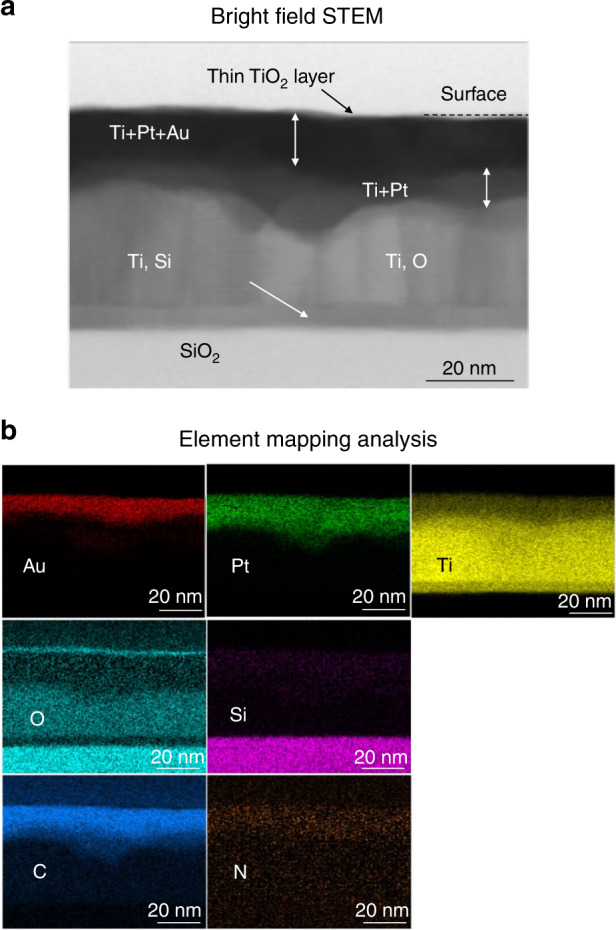
Nanostructure of Au/Pt/Ti film annealed in package. **a** Cross-sectional STEM observation and **b** element mapping analysis using EDS of the Au (3 nm)/Pt (5 nm)/Ti (40 nm) layers of the packaged internal cavity. The specimen was annealed at 450 °C for 180 min after bonding ^20^. Copyright (2021) The Japan Institute of Electronics Packaging

Figure 8 shows the cross-sectional STEM image and element mapping analysis using EDS of the inner surface of the annealed package. Ti, Pt, Au, and O atoms were present on the surface. Moreover, the signals of C and N atoms were detected in the surface region. Since Ti can react with various gases, including H_2_O, O_2_, N_2_, CO, CO_2_, and hydrocarbons^3^, gases containing C and N were likely absorbed. However, there is also a possibility of contamination on Au and Pt atoms after cross-sectioning was performed for observation because they are easily covered with organic contaminants. The layer containing Ti and Pt was present under the Ti, Pt, and Au layers. When the Au/Pt/Ti layer was annealed under atmospheric conditions, the XPS result revealed that Pt atoms were observed in the Ti oxide region. It is supposed that the Pt atoms diffused toward the bottom by gas absorption. Moreover, the presence of unreacted Ti atoms under the surface layer was confirmed.

**Table 1:** Result of fine leak test for the packaged cavities

Bonded substrates having cavity	Bonded substrates without cavity
Detected He gas: 6.531 × 10^−11^ Pa.m^3^/s	Detected He gas: 7.988 × 10^−11^ Pa.m^3^/s

### Investigating gas pressures and species in the package

The gas pressures and species in the packages fabricated using the bonding of Au/Pt/Ti layers were investigated. Under three different conditions, Au/Pt/Ti layers with respective thicknesses of 3/5/40 nm on wafers were bonded in a vacuum under a pressure of ~1 × 10^−2^ Pa:

(A) Bonding at room temperature.
(B) Degas annealing and bonding at 200 °C.
(C) Degas annealing and bonding at 200 °C and annealing at 450 °C for getter activation.

The packages were placed in a vacuum chamber and annealed at 100 °C for 30 min and then broken under He flow (50 mL/min), and the released gases were analyzed via mass spectrometry (GC/MS QP2010 Ultra). This experiment was performed 24 days after the samples were packaged. The measurable detection limit of mass spectrometry is ~50 nL; however, it depends on the gas species. Sixteen packages with lengths, widths, and heights of 10 mm, 10 mm, and 100 µm, respectively, were fractured at the same time; the total volume of the package was 160 µL. The detected gas pressures and species are listed in Table 2. Gaseous carbon species were detected in the samples bonded at room temperature. The amounts of CO_2_ and organic gases were below the detection limit, indicating that these gases were mostly removed by degassing. Moreover, N_2_ gas was detected even in the degassed package but not after the high-temperature annealing process. This result suggests that the diffusion of the Ti underlayer to the internal surface effectively absorbs the residual gas in the package. However, we cannot rule out the possibility that the N_2_ gas in the package was due to gas leakage from atmospheric air and that the high-temperature processes decreased the leak rate.

**Table 2:** Gas pressure and species in the packages fabricated by the bonding of Au/Pt/Ti layers

Mass/charge ratio	Attribution	(A) Bonding at room temperature	(B) Degassing and bonding at 200 °C	(C) Degassing and bonding at 200 °C, and annealing at 450 °C
18	H_2_O	Not detected	Not detected	Not detected
28	N_2_ and so on	2.5 µL	0.042 µL	Not detected
44	CO_2_ and so on	0.0057 µL	Not detected	Not detected
57	Organic gas	0.13 µL	Not detected	Not detected

## Discussion

When the Au and Pt layers were too thin, the Au/Pt/Ti layers could not directly bond after the degas annealing step at 200 °C. When the Au (3 nm)/Pt (5 nm)/Ti (40 nm) multilayer was deposited, the substrates underwent direct bonding after the degassing step. Moreover, by annealing at 450 °C, the Ti underlayer atoms diffused through the Au and Pt layers to the surface and reacted with the gas molecules. The Au/Pt/Ti layer that was annealed under atmospheric conditions consisted mainly of TiO_2_. In addition, the gas pressure analysis summarized in Table 2 suggested that N_2_ gas was possibly absorbed by diffused Ti atoms. However, it was not detected by XPS and EDS; we believed that the number of N compounds was below the measurement limits (~0.1 at.%). However, it is known that inert gases cannot be removed by the diffusion of Ti atoms^24^. The sample annealed in a vacuum package had a Ti composite layer with Ti, Ti^2+^, Ti^3+^, and Ti^4+^ on the surface and a pure Ti layer underneath. The investigation of the gas pressures and species indicated that the diffusion of Ti atoms effectively removed the residual gases in the package. This study demonstrates a packaging process using the bonding of the Au/Pt/Ti layers; however, it is believed that the cap wafer coated with the Au/Pt/Ti layer is applicable to the packaging of other structures (i.e., a MEMS device wafer with Au rings)^25^. Thus, the proposed method is expected to contribute to the simplification of the MEMS structure and manufacturing process because a cap wafer metalized with the Au/Pt/Ti layer can achieve vacuum packaging without any additional deposition and patterning processes.

## Materials and methods

Metal multilayers were deposited on Si substrates 4 inches in diameter using a sputtering system (SME-200E, ULVAC); these samples were used in the following experiments. The Si substrates had a thermally grown SiO_2_ layer of 300 nm thickness on the surface to minimize the diffusion of Si atoms into the metal layer.

### Optimizing the Pt layer thickness

The Pt/Ti layer was deposited on the Si substrates. While the thickness of the Ti layer was set at 40 nm, Pt layers of 2.5 and 5 nm thickness were deposited on the Ti layer to investigate their diffusion barrier ability. In a previous study^5^, the degassing step by vacuum annealing (at 200 °C for 10 min) efficiently removed the absorbed water molecules; thus, the packaged cesium gas cell was successfully achieved. The Pt/Ti layers were degassed under the same conditions, as shown in Fig. 1a.

### Optimizing the Au layer thickness

A pair of Si substrates were metalized with Au (1, 3, and 12 nm)/Pt (5 nm)/Ti (40 nm) layers. The contaminants on the Au surfaces were removed by Ar plasma irradiation in the bonding system (WAP-1000, Bondtech). Subsequently, these substrates were introduced into a vacuum bonding chamber, where they were annealed at 200 °C for 10 min in a vacuum of 1 × 10^−2^ Pa for degassing. Furthermore, the Au surfaces were in contact with each other to initiate atomic diffusion across the bonding interface under a bonding pressure of 123 kPa (1000 N) at 200 °C, as shown in Fig. 1b. Moreover, the Au/Pt/Ti layers were annealed at 400 and 450 °C under atmospheric conditions to induce gas absorption by the diffused Ti atoms.

### Absorbing residual gas molecules in a package

Cavities 10 mm^2^ and ~100 μm deep were fabricated on the Si substrate via alkali etching. The substrate with the cavity was bonded with a flat Si substrate using the Au/Pt/Ti layer (3/5/40 nm in thickness). As shown in Fig. 1c, the substrate surfaces were coated with the Au/Pt/Ti layer, cleaned with Ar plasma, degassed at 200 °C for 10 min in a vacuum, and bonded under a pressure of 123 kPa at 200 °C. The package between the substrates was annealed at 450 °C for 180 min to initiate a reaction between the Ti atoms and gas molecules in the package.

### Evaluation

The quality of bonding was assessed using SAM and a tensile tester. The chemical composition of the surfaces was investigated using X-ray photoelectron spectroscopy (XPS). The compositional structure of the annealed Au/Pt/Ti layer was investigated using scanning transmission electron microscopy (STEM) and energy-dispersive X-ray spectroscopy (EDS). The internal gas pressure and species in the packages were evaluated by breaking the packages into an ultrahigh vacuum chamber with a temperature-programmed desorption mass spectrometry (TPD-MS) system.

## Supplementary information

Cavity structure
