# Supplementary figures and images for: Bonding formation and gas absorption using Au/Pt/Ti layers for vacuum packaging
Source: Microsyst Nanoeng. 2022 Jan 17;8:2. doi: 10.1038/s41378-021-00339-x (PMC8761747; doi:10.1038/s41378-021-00339-x)

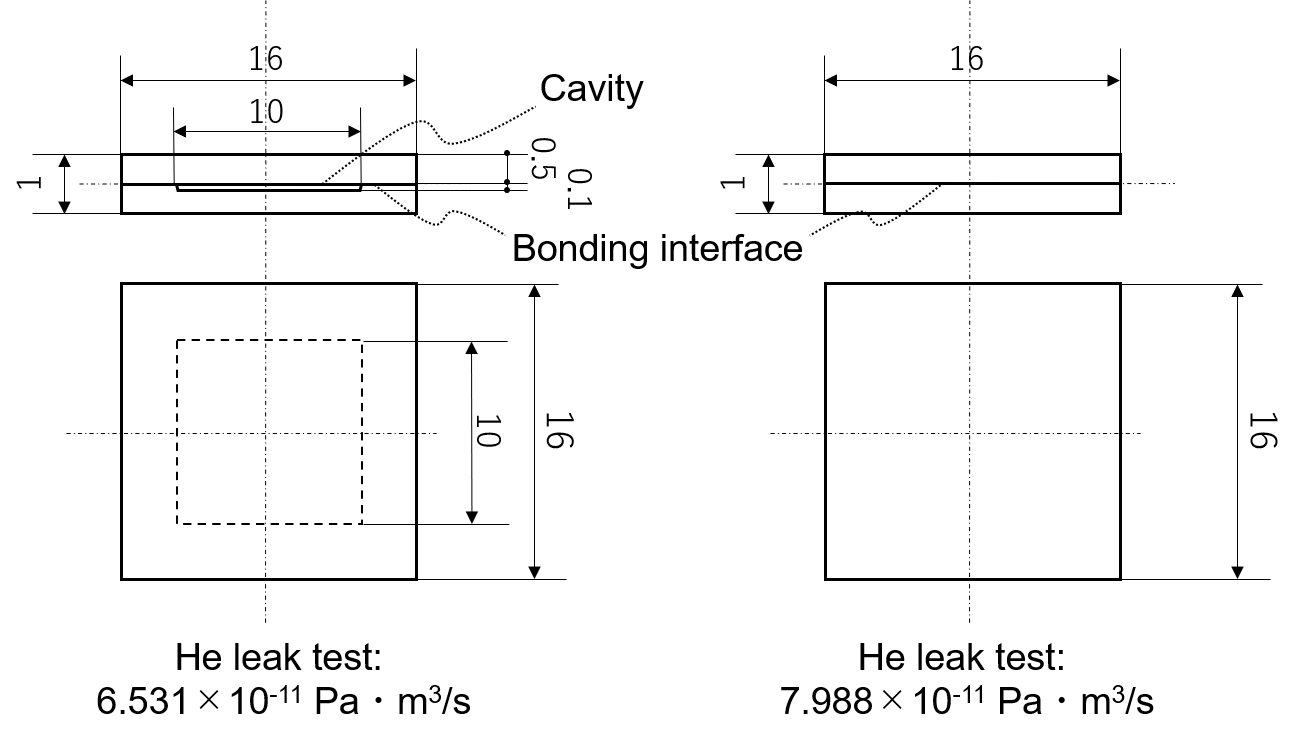

Supplement: Supplementary file 1 — Cavity structure [file 41378_2021_339_MOESM1_ESM.tif]
